# Caspr3-Deficient Mice Exhibit Low Motor Learning during the Early Phase of the Accelerated Rotarod Task

**DOI:** 10.1371/journal.pone.0147887

**Published:** 2016-01-25

**Authors:** Haruna Hirata, Aki Takahashi, Yasushi Shimoda, Tsuyoshi Koide

**Affiliations:** 1 Department of Bioengineering, Nagaoka University of Technology, Nagaoka, Niigata, 940–2188, Japan; 2 Mouse Genomics Resource Laboratory, National Institute of Genetics (NIG), Mishima, Shizuoka, 411–8540, Japan; 3 Department of Genetics, The Graduate University for Advanced Studies (SOKENDAI), Mishima, Shizuoka, 411–8540, Japan; 4 Transdisciplinary Research Integration Center, Research Organization of Information and Systems, Minato-ku, Tokyo, 105–0001, Japan; 5 Laboratory of Behavioral Neuroendocrinology, University of Tsukuba, Tsukuba, Ibaraki, 305–8577, Japan; Rikagaku Kenkyūsho Brain Science Institute, JAPAN

## Abstract

Caspr3 (Contactin-associated protein-like 3, Cntnap3) is a neural cell adhesion molecule belonging to the Caspr family. We have recently shown that Caspr3 is expressed abundantly between the first and second postnatal weeks in the mouse basal ganglia, including the striatum, external segment of the globus pallidus, subthalamic nucleus, and substantia nigra. However, its physiological role remains largely unknown. In this study, we conducted a series of behavioral analyses on Capsr3-knockout (KO) mice and equivalent wild-type (WT) mice to investigate the role of Caspr3 in brain function. No significant differences were observed in most behavioral traits between Caspr3-KO and WT mice, but we found that Caspr3-KO mice performed poorly during the early phase of the accelerated rotarod task in which latency to falling off a rod rotating with increasing velocity was examined. In the late phase, the performance of the Caspr3-KO mice caught up to the level of WT mice, suggesting that the deletion of Caspr3 caused a delay in motor learning. We then examined changes in neural activity after training on the accelerated rotarod by conducting immunohistochemistry using antibody to c-Fos, an indirect marker for neuronal activity. Experience of the accelerated rotarod task caused increases in the number of c-Fos-positive cells in the dorsal striatum, cerebellum, and motor cortex in both Caspr3-KO and WT mice, but the number of c-Fos-positive cells was significantly lower in the dorsal striatum of Caspr3-KO mice than in that of WT mice. The expression of c-Fos in the ventral striatum of Caspr3-KO and WT mice was not altered by the training. Our findings suggest that reduced activation of neural cells in the dorsal striatum in Caspr3-KO mice leads to a decline in motor learning in the accelerated rotarod task.

## Introduction

Contactin-associated protein-like 3 (Caspr3, also known as Cntnap3) is a neural cell adhesion molecule belonging to the Caspr family, which comprises five members: Caspr, Caspr2, Caspr3, Caspr4, and Caspr5 [1–4]. Caspr and Caspr2 play essential roles in the formation and maintenance of myelinated nerves via interaction with Contactin and TAG-1, respectively, belonging to the Contactin family [5–7]. Null mutant mice of Caspr exhibit tremor, ataxia and significant motor paresis, and die within a month [8]. Mice lacking Caspr2 display epilepsy and autism-related deficits, such as abnormal vocal communication, repetitive and restrictive behaviors, and abnormal social interactions [9]. It was also reported that Caspr2 knockdown leads to decreases in dendritic arborization and spine development [10]. In addition, it has been shown that Caspr4 plays essential roles in synaptic transmission in cortical interneurons; mutant mice exhibit excessive grooming and increases in startle response amplitude and prepulse inhibition [11]. By contrast, the function of Caspr3 remains largely unknown.

We previously generated Caspr3-knockout (KO) mice and monoclonal antibodies against Caspr3, and have reported that Caspr3 is expressed abundantly between the first and second postnatal weeks in mouse basal ganglia, including the striatum, external segment of the globus pallidus, subthalamic nucleus, and substantia nigra [12]. In the striatum, the primary input nucleus of the basal ganglia, Caspr3 was shown to be expressed in cholinergic interneurons and a subpopulation of striatal projection neurons. However, Caspr3 KO mice have no gross abnormalities in the basal ganglia, as shown by Nissl staining [12]. Given that the basal ganglia have very complicated structures and are involved in motor control and learning, their dysfunction causes movement disorders such as Parkinson’s disease, Huntington’s disease, attention deficit hyperactivity disorder, and hemiballism [13–16]. Based on these studies, it is assumed that Caspr3 plays a role in regulating motor control or learning behavior.

In this study, we explored whether Capsr3-KO mice exhibit any behavioral abnormalities using a battery of 11 different behavioral tests including tasks associated with motor control and learning. Our results showed that Caspr3-KO mice displayed delayed motor learning in the accelerated rotarod task. We then examined the expression of c-Fos, an indirect marker for neuronal activity, in several brain regions in wild-type (WT) and Caspr3-KO mice after training on the rotarod and found a reduction in the number of c-Fos-positive cells in the dorsal striatum of Caspr3-KO mice.

## Results

### Aberration of Caspr3 leads to delayed motor learning in the accelerated rotarod task

To investigate the effect of null mutation in Caspr3, we conducted a series of behavioral tests (Table 1, S1–S4 Tables). As Table 1 shows, no significant differences between WT and Caspr3-KO mice were detected in any of the behavioral tests except for the accelerated rotarod test.

**Table 1:** Summary of results from the behavioral test battery. WT and Caspr3-KO mice showed no significant differences in spontaneous activity, anxiety, working memory, aggressive behavior, fear learning, startle response, prepulse inhibition (PPI) or pain sensitivity. A significant behavioral difference between WT and Caspr3-KO mice was observed only in the accelerated rotarod test (see Fig 1). All data are presented as mean ± SEM.

Schedule	Test	Index	WT	n	Caspr3 KO	n
Days 1–4	Home cage test	Total activity	44,853.1 ± 3,303.5	17	41,021.7 ± 2,364.3	17
		Active time (min)	1,550.7 ± 62.9	17	1,529.1 ± 52.4	17
Day 5	Open field test	Total distance (cm)	5402.0 ± 218.5	13	5,423.6 ±183.7	12
		Total time in central area (s)	7.3 ± 1.0	13	5.3 ± 0.8	12
		Locomotion (s)	272.9 ± 10.7	13	276.4 ± 10.0	12
		Leaning (s)	71.0 ± 9.8	13	81.9 ± 9.3	12
		Rearing (s)	37.1 ± 6.9	13	29.5 ± 5.4	12
		Grooming (s)	11.8 ± 1.7	13	11.2 ± 1.8	12
		Pausing (s)	5.5 ± 1.8	13	11.5 ± 4.9	12
		Stretching (s)	1.3 ± 0.5	13	3.0 ± 1.2	12
Day 6	Elevated plus maze test	Total distance (cm)	124.1 ± 7.7	13	118 ± 7.9	12
		Total time in open arm (s)	15.1 ± 5.5	13	12.3 ± 4.8	12
Day 7	Y-maze test (spontaneous alternation)	% alternation	60.3 ± 6.2	13	68.5 ± 3.5	12
Days 8–12	Accelerated rotarod test	Time (s)	See Fig 1		See Fig 1	
		Learning rate	See Fig 1		See Fig 1	
Days 13–14	Resident–intruder test	Nonaggressive behaviors (s)	232.0 ± 29.1	12	221.7 ± 20.5	12
		Aggressive behaviors (s)	117.0 ± 48.8	12	156.5 ± 44.6	12
Day 15	Y-maze test (working memory)	% first choice norvel arm	75	12	75	12
		No. of norvel arm entries	5.5 ± 0.8	12	5.6 ± 0.6	12
		Total time in norvel arm (s)	95.5 ± 11.5	12	81.7 ± 6.0	12
Days 16–18	Fear conditioning test	Time of freezing (s)	101.5 ± 18.4	12	108.2 ± 14.2	12
Day 19	Startle response and PPI test	Startle magnitude	488.7 ± 78.1	12	447.3 ± 60.8	12
		Response time (ms)	21.4 ± 1.5	12	20.5 ± 1.8	12
		PPI rate	43.8 ± 3.9	12	35.6 ± 6.2	12
		prepulse response time (ms)	21.5 ± 1.7	12	21.4 ± 1.0	12
Day 20	Hot plate test	Time (s)	23.9 ± 1.5	12	23.1 ± 1.2	12
Day 21	Tail flick test	Time (s)	2.6 ± 0.2	12	2.6 ± 0.2	12

**Fig 1:**
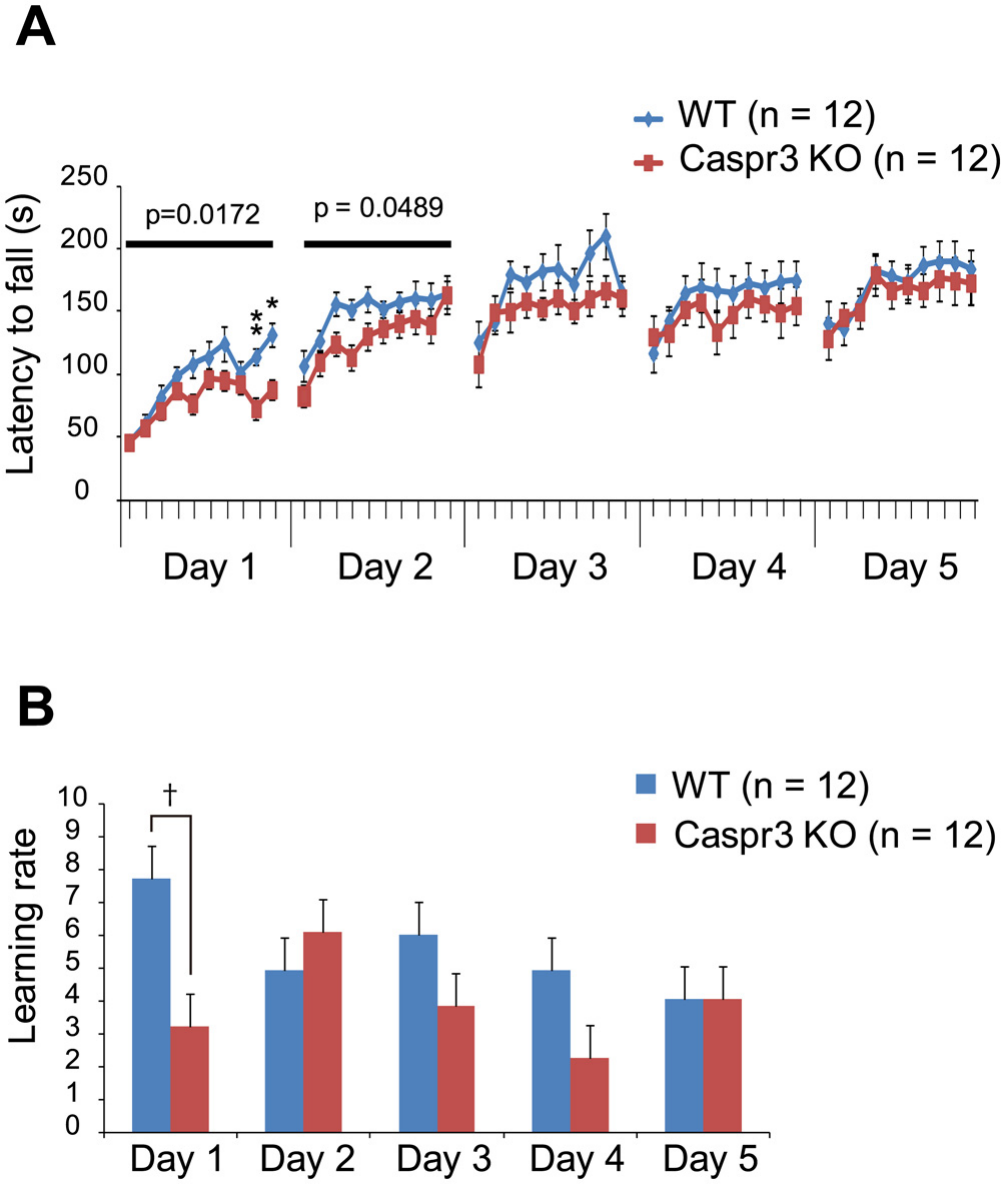
Caspr3-KO mice showed low motor learning during the early phase of the rotarod task. [A] Latency to fall of WT and Caspr3-KO mice during training on the accelerated rotarod for five consecutive days. The performance of Caspr3-KO mice on the first two training days was significantly worse than that of WT mice. Repeated-measures two-way ANOVA was conducted to examine the main effect of genotype on each day (p values indicated). [B] Learning rate of WT and Caspr3-KO mice during training on the accelerated rotarod. The learning rate of Caspr3-KO mice on the first training day was significantly worse than that of WT mice. *p < 0.05, **p < 0.01 by *t*-test with Bonferroni correction. ^†^p < 0.05 by Mann-Whitney test with Bonferroni correction (number of comparisons was ten for latency to fall, and five for learning rate). n = 12 mice per genotype. Error bars represent SEM.

The accelerated rotarod test was performed to evaluate motor coordination and motor learning (Fig 1; S3 and S4 Tables). In this test, motor performances of mice were analyzed by measuring latency to falling off a rod that was rotated with increasing velocity. Animals were trained in 10 trials per day for five consecutive days on a rotating rod. WT mice showed marked improvements in their performance during the first 2 days, and the performance reached a plateau on the third day (Fig 1A). The motor performance was maintained after the fourth day. In contrast, the performances of Caspr3-KO mice on days 1 and 2 were significantly worse than those of the WT mice (day 1: F(1, 22) = 6.642, p = 0.0172; day 2: F(1, 22) = 4.346, p = 0.0489; Fig 1A). Especially on day 1, Caspr3-KO mice showed significantly shorter latency to fall than WT mice in trial 9 (WT: 114 ± 6.2 s, Caspr3 KO: 73 ± 8.7 s, p = 0.0080) and trial 10 (WT: 131.8 ± 9.9, KO: 88.0 ± 8.0 s, p = 0.0230) (Fig 1A). No significant differences were detected in the performance on days 3, 4, and 5 between the two genotypes. We also calculated the learning rate on each day (Fig 1B) and found that the learning rate of the Caspr3-KO mice on day 1 was significantly lower than that of the WT mice (p = 0.0100; Fig 1B). There was no significant difference in the learning rate on day 2 between WT and Caspr3-KO mice, even though the performance on day 2 was significantly different between the two genotypes. Similarly, there was no significant difference in the learning rates on days 3, 4, and 5 between WT and Caspr3-KO mice. These findings suggested that the knockout of Caspr3 influences motor learning during the early phase of the accelerated rotarod task, but does not affect motor performance in the later phase of the learning task.

**Fig 2:**
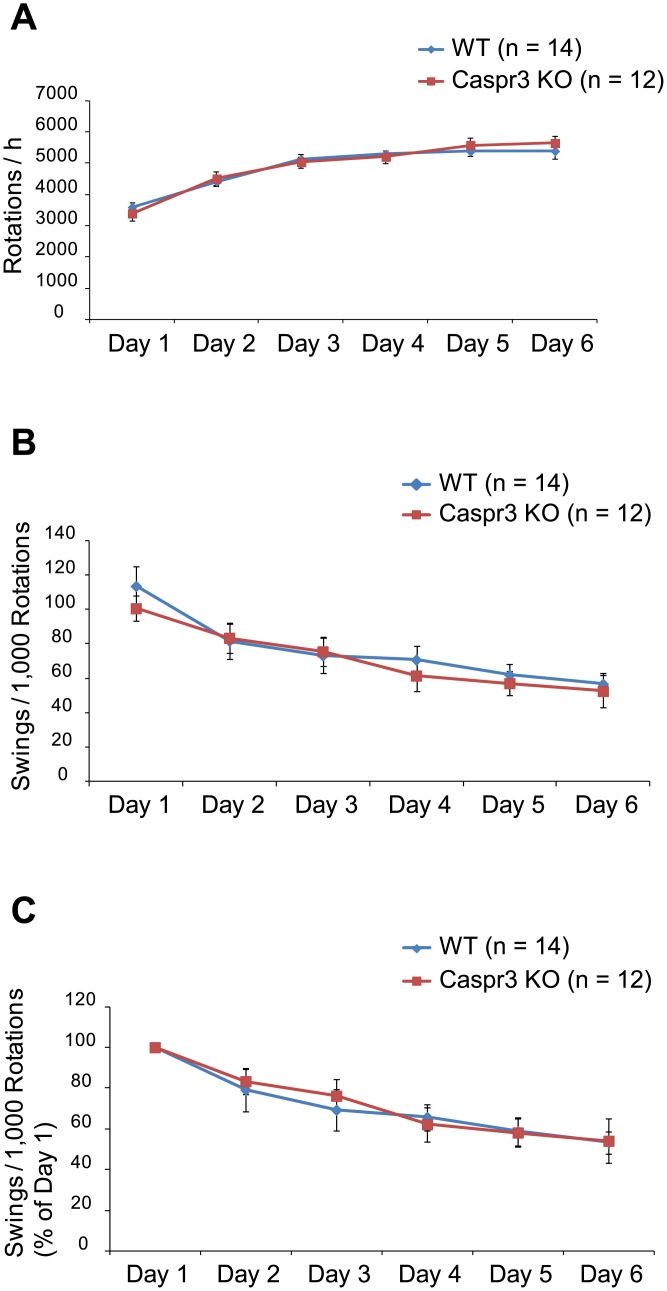
Assessment of motor performance using wheel running apparatus. [A] Total number of wheel rotations per hour upon wheel running for six consecutive days. The number of wheel rotations gradually increased but showed no significant difference between WT and Caspr3-KO mice. [B] Number of running errors (swings) per 1,000 rotations during wheel running training. No significant differences were observed in the numbers of running errors between WT and Caspr3-KO mice. [C] The number of running errors per 1,000 rotations, expressed as a percentage of the error score on day 1, on days 2–6. The errors of WT and Caspr3-KO mice decreased during the days of training, and there was no significant difference between the two genotypes. Error bars represent SEM. n = 14 (WT mice), n = 12 (Caspr3-KO mice).

Next, we conducted the wheel running test to measure motor learning, which differs from the accelerated rotarod test (S5 and S6 Tables). Animals were trained for 1 h per day for six consecutive days inside a wheel. For WT and Caspr3-KO mice, the number of wheel rotations gradually increased but showed no significant difference between the two genotypes (Fig 2A). During the training, mice were occasionally swung around inside the wheel due to running errors. We counted the number of errors (swings) during a 1-h session by video analysis. The motor learning abilities were estimated by calculating the errors per 1,000 rotations (Fig 2B and 2C). No significant difference between WT and Caspr3-KO mice was detected in the number of errors per 1,000 rotations on any day.

### Decrease of c-Fos activity in the dorsal striatum after training on the rotarod in Caspr3-KO mice

**Fig 3:**
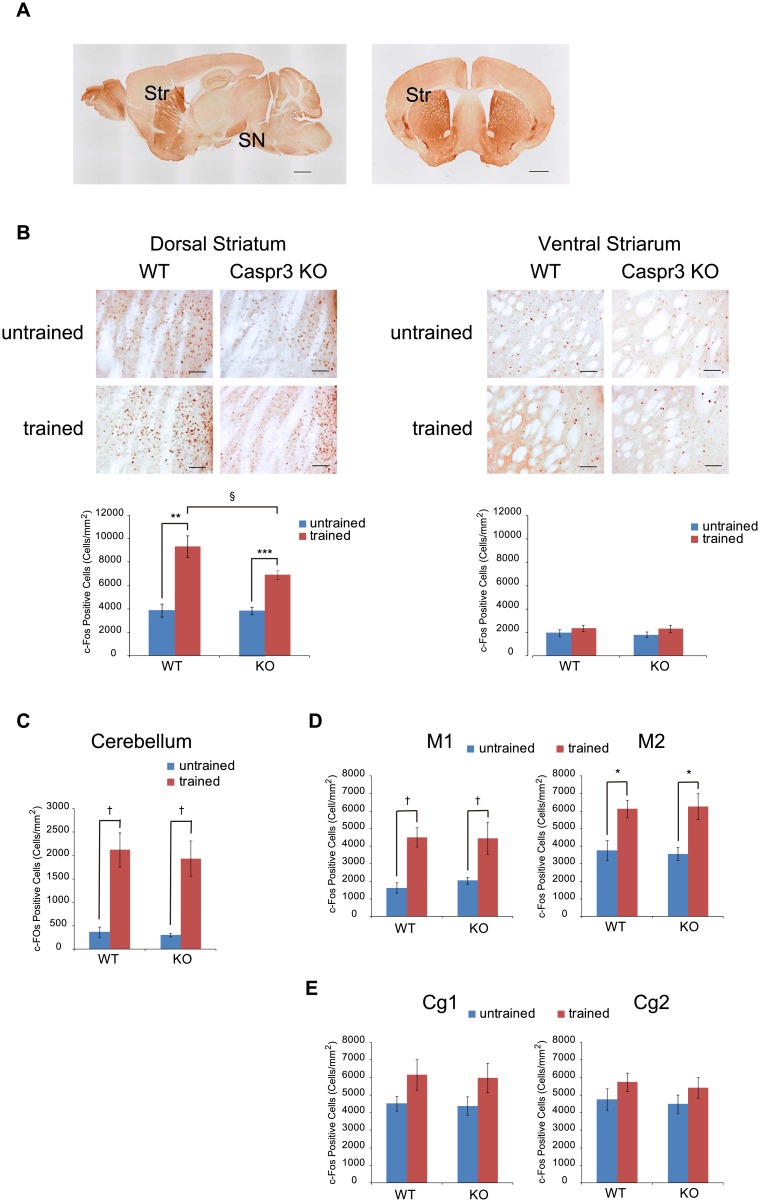
Quantification of c-Fos-expressing cells in WT and Caspr3-KO mice after training on the accelerated rotarod. [A] Localization of Caspr3 in adult WT mice. Caspr3 was strongly expressed in the striatum (Str) and substantia nigra (SN). Expression of Caspr3 in the striatum was stronger in the medial area than in the lateral area. Scale bars: 1 mm. [B–E] Comparison of the number of c-Fos-expressing cells in several brain regions between WT and Caspr3-KO mice after training in the accelerated rotarod task. Significant increases in the number of c-Fos-positive cells were observed in the dorsal striatum of WT and Caspr3-KO mice after the training. However, the number of c-Fos-positive cells after the training was lower in Caspr3-KO mice than in WT mice [B]. Scale bars: 100 μm. The number of c-Fos-positive cells in the ventral striatum was not significantly different between the two genotypes [B]. In the cerebellum [C] and motor cortex [D], c-Fos-positive cells were increased after the training in both WT and Caspr3-KO mice, but there was no significant difference between the two genotypes. No significant differences were detected in the number of c-Fos-positive cells in the cingulate cortex [E] between the two genotypes. ^§^p < 0.10, *p < 0.05, **p < 0.01, ***p < 0.001 by *t*-test with Bonferroni correction (number of comparisons was four). ^†^p < 0.05 by Mann-Whitney test with Bonferroni correction. n = 6 untrained mice per genotype; n = 6 trained WT mice; n = 7 trained Caspr3-KO mice. Error bars represent SEM.

In the adult mouse brain, strong signals from Caspr3 were observed mainly in the striatum and substantia nigra, and the expression of Caspr3 in the striatum was greater in the medial area than in the lateral area (Fig 3A). To examine whether delayed motor learning in the accelerated rotarod task in Caspr3-KO mice is due to reduced neural activity in the brain area where Caspr3 is detected, we conducted immunohistochemistry analysis in several brain areas using c-Fos antibody, which indirectly detects cells exhibiting neuronal activity, after training on the accelerated rotarod (Fig 3B–3E, S7 and S8 Tables, all the image files have been deposited in the Dryad database: doi:10.5061/dryad.tt550). In the dorsal striatum, two-way ANOVA showed significant main effects of genotype (F(1, 21) = 5.581, p = 0.0279) and training (F(1, 21) = 66.736, p < 0.0001), as well as genotype x training interaction (F(1, 21) = 5.253, p = 0.0323; Fig 3B). Both Caspr3 WT and KO mice exhibited a significant increase in c-Fos-positive cells in the dorsal striatum after training (WT: n = 6 each; p = 0.0008; Caspr3-KO: n = 6 untrained and 7 trained; p < 0.0001; Fig 3B). However, the increase in c-Fos-positive cells by training was lower in Caspr3-KO mice than in WT mice, and there was a difference between WT and Caspr3-KO after training (p = 0.0596; Fig 3B). No increase of c-Fos expression after training was observed in the ventral striatum in either WT or Caspr3-KO mice (Fig 3B). An effect of training, but not of genotype, was observed in the cerebellum, motor cortex M1, and motor cortex M2. Significant increases in c-Fos-positive cells were detected after training on the rotarod in the cerebellum (WT: p = 0.0156; Caspr3-KO mice: p = 0.0108) and motor cortex M1 (WT: p = 0.0156; Caspr3 KO: p = 0.0264), but there was no significant difference between the two genotypes (Fig 3C and 3D). Similarly, only the effect of training was significant in motor cortex M2 (F(1, 21) = 24.463, p < 0.0001), and post hoc tests indicated significant increases of c-Fos-positive cells after training in both WT (p = 0.0268) and Caspr3 KO (p = 0.0164; Fig 3D) mice. In the cingulate cortex Cg1 and Cg2, neither a significant increase of c-Fos-positive cells after training nor a significant genotype difference was observed (Fig 3E).

Collectively, these results suggest that reduced motor learning in the accelerated rotarod task in Caspr3-KO mice was caused by a reduced activation of neural cells in the dorsal striatum.

## Discussion

### Behavioral phenotype of Caspr3-KO mice

To understand the role of Caspr3, we analyzed the behavioral phenotypes of Caspr3-KO mice and their WT littermates. Caspr3-KO mice exhibited no difference from WT mice in terms of spontaneous activity, anxiety-like behaviors, and nociception. In the cases of Caspr2- and Caspr4-KO mice, different levels of social behavior, grooming, and prepulse inhibition (PPI) were previously observed compared with WT mice [9, 11]. However, in the present study, no differences in these behaviors were observed between Caspr3-KO and WT mice. In contrast, we found that Caspr3-KO mice showed reduced motor learning in the accelerated rotarod task. The accelerated rotarod task has been widely used to study motor learning [17–21]. However, Caspr3-KO mice did not show any change of working memory in the Y-maze test or of fear learning in the fear conditioning test. These results indicate that Caspr3 plays an important role in motor learning but not in fear learning or working memory.

### The role of Caspr3 in motor learning

It has been reported that motor learning is divided into two phases, “fast” and “slow” learning. In fast learning, a great improvement in motor performance is observed within the first session, followed by a period of consolidation for several hours. In slow learning, motor performance is improved across sessions [22]. In previous studies of motor learning using the accelerated rotarod task, animals showed improvement of performance during training on day 1, and exhibited less improvement on the second and third training days [18, 23]. A study using multielectrode arrays revealed that the neuronal ensemble activity in the motor cortex and dorsal striatum changed through the different phases of learning [18]. Another study showed that neural activity in the dorsomedial striatum is preferentially engaged in the early phase of training, and the neural activity in the dorsolateral striatum is associated with the late phase of training [24].

It has been demonstrated that neural activity was suppressed by Caspr2 knockdown in cultured cortical primary neurons [10]. In the same study, a Caspr3-knockdown experiment was also performed and showed an inhibition of neural activity in the cultured cortical neurons. This indicated that Caspr3 may modulate neural activity in the striatum.

Caspr3 is highly expressed in postnatal developmental stages in the basal ganglia, such as the striatum, nucleus accumbens, external segment of the globus pallidus, and substantia nigra [12]. In postnatal stages, cell adhesion molecules play important roles in developmental events such as myelinations and synaptogenesis throughout the brain [5–7, 25–27]. It has been reported that neural maturation was observed for morphological and physiological characteristics in the striatum during the second postnatal week [28–31]. Although Nissl staining in histological analyses showed that Caspr3 KO mice have normal morphological structures in the basal ganglia [12], it is possible that undetectable changes in neural maturation might be caused by the lack of Caspr3 during postnatal development. Given that the protein product CASPR3 is localized in striatonigral fibers [12], it will be important to determine whether Caspr3 KO mice have normal circuit formation in the basal ganglia.

In the adult mouse brain, Caspr3 was observed in the striatum and substantia nigra, and higher expression of Caspr3 was detected in the medial area of the striatum than in the lateral area. The striatum and motor cortex are critical for motor learning [18, 32, 33]. Furthermore, it has been reported that the expression of c-Fos, a neural activation marker, was increased in the dorsal striatum, cerebellum, and cingulate cortex after training for the first 10 trials in the accelerated rotarod task [19]. In the present study, Caspr3 KO mice showed fewer c-Fos-positive cells in the dorsal striatum after the first training session on the accelerated rotarod task. These results suggest that Caspr3 may be associated with the control of neural activity in the dorsomedial striatum, leading to the regulation of motor learning on the rotarod task.

In contrast, Caspr3 deficiency did not significantly impact motor learning in the wheel running test. The rotarod test is a forced motor task, whereas the wheel running test, in which mice run freely during the test period, involves a focus on different behavioral properties to measure motor learning. Wheel running is known to be driven by a motivational factor and is considered to involve reward-related behavior [34]. In addition, reward has positive effects on maintaining or increasing motor learning [35–37]. Previous studies showed that the nucleus accumbens, a ventral striatum subregion, is associated with reward behaviors [38–41]. It has been reported that c-Fos expression was induced not only in the dorsal striatum but also in the nucleus accumbens by wheel running [42, 43]. There is a possibility that the acquisition of a reward by wheel running influenced the improvement in motor learning.

### The relationship between striatal synaptic plasticity and Caspr3

Striatal synaptic plasticity is regulated by a variety of neurotransmitters, including glutamate [44–47]. For example, it has been reported that the N-methyl D-aspartate (NMDA) receptor in the striatum is involved in instrumental learning [48, 49]. NMDA receptors are composed of an obligatory NR1 subunit with different combinations of NR2A-D subunits, which alter channel kinetics [50]. The functional properties of NMDA receptors are largely dependent on the composition of these subunits [51, 52]. NR1, NR2A, and NR2B are expressed preferentially in the striatum [53, 54]. Mice with knockout of the striatum-specific NR1 subunit showed impaired motor learning on the accelerated rotarod task [55], and pharmacologic blockade of the NR2A subunit in the dorsal striatum influenced the late phase of learning in the task [56]. Meanwhile, transgenic mice that overexpress NR2B are known to achieve superior scores in various learning and memory tasks [57]. NR2B interacts with Mint1, an adaptor protein, and the interaction of Mint1 and KIF17, a neuron-specific microtubule-dependent molecular motor, is involved in the transport of vesicles containing NR2B [58]. It is also known that KIF17 is critical to the maintenance of NR2A/2B levels [59]. It has been reported that Caspr3 interacts with Mint1/X11 via the PDZ domain-binding motif at the C-terminus of Caspr3 [3], suggesting that Caspr3 also interacts with NR2B through the binding to Mint1/X11. Caspr (as distinct from Caspr3) interacts with GluA1, a subunit of the α-amino-3-hydroxy-5-methyl-4-isoxazolepropionic acid-type glutamate receptor [60]. Caspr is required for the trafficking of GluA1 to the synapses, and the coexpression of Caspr with GluA1 increases the amplitude of GluA1-mediated currents. Collectively, these reports suggest that Caspr3 is associated with the transportation of NR2B, contributing to motor learning in the rotarod task. However, it remains unclear whether Caspr3 is localized in the synapses, even though punctate signals from Caspr3 were detected throughout the basal ganglia, cortex, and hippocampus [12]. Caspr3-positive signals did not overlap with presynaptic markers such as VGluT1, VGAT, and GAD67, in these brain regions on postnatal day 7, although this is still early in the process of synaptogenesis. We should conduct further detailed analyses using histological double staining with synaptic markers such as VGluT1, VGAT, GAD67, glutamate receptors GABA receptors, and dopamine receptors at postnatal day 14 or later, followed by immunoelectron microscopy analysis and protein analysis of the synaptosomal fraction. These analyses will reveal whether Caspr3 is localized in the synapses. It is also necessary to investigate the interaction of Caspr3 and NR2B in future study in order to understand the role of Caspr3 during motor learning.

## Materials and Methods

### Animals

Caspr3-KO mice were backcrossed to the C57BL/6JJmsSlc (B6) strain for 12 generations and kept as a congenic strain [12]. B6 mice were purchased from Japan SLC (Hamamatsu, Japan). Caspr3-KO homozygote males were mated with B6 females, and F_1_ males and the females were crossed to produce F_2_ mice. Caspr3-KO homozygote (Caspr3-KO) and wild-type (WT) mice were obtained from the F_2_ mice by genotyping at 4 weeks-of-age and kept with littermates of the same sex until used for experiments. Male mice were used for the experiments throughout this study. Behavioral tests were conducted at 10 to 13 weeks-of-age, during which the mice were housed singly. All animals were housed in standard mouse cages (19.5 × 29.5 × 15 cm) with wood shavings at the National Institute of Genetics (NIG) under a 12/12-h light/dark cycle (lights on from 06:00 to 18:00 h) in a humidity- and temperature-controlled room (50 ± 10%, 23 ± 2°C). Food and water were available ad libitum. All behavioral experiments except for the home cage activity test were performed during the light period. Mice were maintained in accordance with NIG guidelines. This study was carried out in strict accordance with the recommendations in the Guidelines for Proper Conduct of Animal Experiments of the Science Council of Japan. The protocol was approved by the Institutional Committee for Animal Care and Use of the National Institute of Genetics (Permit Numbers: 24–10 and 25–10). All perfusion fixations were performed under sodium pentobarbital anesthesia, and all efforts were made to minimize suffering.

### Behavioral test battery

The series of behavioral tests included 11 different tests (Table 1). This battery was designed for the measurement of spontaneous activity, anxiety, social behavior, aggressive behavior, motor function, working memory, fear learning, sensorimotor gating, and nociception. The body weight of each mouse was measured before the test battery. The sequence of the test battery and the number of animals used are shown in Table 1.

### Home cage activity test

Spontaneous activity was recorded with an infrared sensor (Activity Sensor; Ohara Co. Ltd., Tokyo, Japan) for individual mice in a home cage to which they had been habituated, as described previously [61]. The activity was measured for 4 days; the first day was used for habituation. The total activity score was calculated as the total of activities for each minute over the 3 remaining days. Active time was calculated as the total number of minutes in which the mouse accumulated more than one count within a 1-min interval.

### Open field test

The open field test was conducted in accordance with a method described previously [62, 63]. The open field used consisted of a square arena (60 × 60 × 40 cm) made of a white polyvinylchloride plastic board. The arena was brightly lit by incandescent lighting (365 lux). To analyze overall ambulation distance as well as time spent in the center, the arena was recorded continuously with a video camera placed over its center and the footage was relayed to a video tracking system (Image OF; Ohara Co. Ltd.) based on the National Institutes of Health (NIH) ImageJ software. Animals were placed in the arena for 10 min. During the test, we observed their behavior directly and scored the presence or absence of six behavioral items using the freeware tanaMove, Version 0.07 (http://www.nig.ac.jp/labs/MGRL/tanaMove.html).

Locomotion: walking and running around the arena.

Leaning: standing on the hindlimbs with the forelimbs against the wall.

Rearing: standing on the hindlimbs without touching the wall.

Grooming: licking and/or scratching the fur, licking the genitalia and tail.

Pausing: a brief moment of inactivity.

Stretching: stretching the whole body forward while keeping the hindlimbs in place.

### Elevated plus maze test

The elevated plus maze test was used to assess anxiety-like behaviors, on the basis of a previous report [63]. The apparatus, made of a white acrylic board, consisted of two closed arms enclosed by a clear acrylic plastic wall (30 × 5 × 15 cm) and two open arms without a wall (30 × 5 × 0.25 cm) that extended from a central platform (5 × 5 cm). It was elevated 60 cm above the floor and was dimly lit (150 lux). Mice were placed on the central platform and allowed to explore freely for 10 min. Ambulatory activity, the numbers of entries into the open and closed arms, and durations in the open and closed arms were measured using a video tracking system (Image EPM; Ohara Co. Ltd.) based on the NIH ImageJ software.

### Y-maze test

The Y-maze test was performed according to a method described previously [64]. The Y-maze apparatus (Ohara Co. Ltd.) consisted of three identical arms (37.5 × 3.5 × 12 cm) radiating at 120° angles from a central platform. It was dimly lit (150 lux). We assessed spontaneous alternation of entries into the three arms and working memory, as described below. We set a 7-day interval between the evaluations of spontaneous alternation and working memory in order to avoid the influence of a failure to acclimate to the apparatus and anxiety, in accordance with a previous study [53]. Entry into an arm was defined as occurring when more than one-third of the mouse’s body had entered the arm. All tests were recorded with a video camera and analyzed by an observer using tanaMove.

Spontaneous alternation: All three arms were open. Mice were placed in one of the arms and allowed to explore freely for 6 min. The number and sequence of arm entries were recorded. The percentage of alternations was calculated as follows: % alternations = [number of alternations] / [total possible alternations] × 100.

Working memory: One of the arms was determined as the start arm, and one of the arms was closed. Mice were placed into the start arm and allowed to explore the maze for 15 min (training trial). After a 1-h intertrial interval, all arms were opened. Mice were returned to the start arm and allowed to explore freely for 5 min (test trial). In the test trial, the number of entries into and the time spent in each arm, and the first choice of entry, were observed from video recordings by an observer using tanaMove.

### Accelerating rotarod test

Motor coordination and learning were measured by the accelerating rotarod test, similarly to the approaches described in our previous report [65] and elsewhere [20]. The apparatus, which consisted of a black striated rod (3 cm in diameter) 20 cm above the floor (Ohara Co. Ltd.), was set to accelerate from 6 to 60 rpm over a 300-s period. Animals were placed on the rod with constant low-speed rotation (6 rpm). After the mouse had got used to walking on the rod, the test was started. The time until the mouse fell from the rod was measured automatically. Mice were trained for five consecutive days, with one daily session consisting of 10 trials separated by 300-s resting periods. The learning rate in each session was calculated as follows: learning rate = (mean latency to fall_trials 9 and 10_) − (mean latency to fall_trials 1 and 2_) / (number of intertrial intervals [which was 9 in this study]).

### Resident–intruder test

Aggressive behaviors were observed using the resident–intruder test. C57BL6/J mice (maintained at the NIG) that were younger than the subject males were used as intruders. An intruder mouse was placed into the home cage of the subject (resident) mouse, and the aggressive (attack bites and aggressive grooming) and nonaggressive social behaviors (sniffing and following) of the resident mouse were observed. The observation was performed for two consecutive days for 15 min a day, and the data were summed. All tests were recorded with a video camera and analyzed by an observer using tanaMove.

### Fear conditioning test

The fear conditioning test was used to assess fear learning. All of the conditioning procedures were conducted in a conditioning chamber (30 × 10 × 15 cm, Ohara Co. Ltd.) for two consecutive days. The conditioning stimulus (CS) consisted of a sound (1 kHz, 55 dB) for 4 s, preceding the unconditioned stimulus (US; electric stimulation: 75 V constant, ≤0.3 mA, through the grid floor) by 1 s. The next day, mice were placed into a new cage without wood shavings and habituated for 1 h. They were exposed to the CS sound for 5 min without the US. All tests were recorded with a video camera, and the duration of freezing was measured by an observer using tanaMove.

### Startle response and PPI test

Acoustic startle response and its PPI were measured in accordance with a method described previously [65]. An apparatus with a startle chamber consisting of a clear acrylic tube (San Diego Instruments, San Diego, CA) was used for the tests. The startle response was recorded in the presence of a 65-dB white noise background. All PPI test sessions consisted of 38 trials, including habituation trials. The startle trials (pulse-only) consisted of a 40-ms, 120-dB pulse of broad-band noise. The prepulse trials consisted of a 20-ms prepulse sound (70, 75, 80 dB) followed by a 30-ms, 120-dB sound pulse with a 70-ms interval. Each trial was presented six times, and the average amplitude was calculated. PPI rates were calculated as follows: inhibition rate = (1 − [prepulse + pulse] / [pulse-only]) × 100. The mean values of PPI in all prepulse conditions were used for statistical analysis.

### Hotplate test

A hotplate apparatus (model MK-350A, Muromachi Kikai Co., Ltd., Tokyo, Japan) was used to assess pain sensation, as described previously [66]. The hotplate surrounded by a wall made of acrylic acid resin was heated and maintained at 52°C during the test. Mice were placed on it and the time that they took to lick or shake the hindlimb was measured by an observer. If mice jumped during the test, it was considered to be a response to the pain sensation.

### Tail flick test

Using an apparatus consisting of a radiant heat source and a photosensor for detection of the tail flick (model MK-330A, Muromachi Kikai Co., Ltd.), response latencies were measured as described previously [66]. Mice were held gently on the apparatus, and the ventral surface of the tail was exposed to radiant heat. Latency to the response to the heat stimulus was recorded automatically with an infrared sensor three times per individual at three different positions on the tail. The median value was used for statistical analysis.

### Wheel running test

The wheel running test was used to assess motor learning, as described by Willuhn and Steiner [42]. The running wheels (Ohara Co. Ltd.) consisted of a rotating metal chamber with a wire mesh floor (15 cm in diameter, 5 cm wide), attached to a stationary metal wall. Mice were trained on a running wheel for 60 min on six consecutive days. During the training session, the animal was free to run, but could not leave the wheel. Numbers of rotations were measured by a mechanical counter (Ohara Co. Ltd.). A state in which the mouse slipped and was swung like a pendulum was considered to be a running error. All tests were recorded with a video camera, and the numbers of errors were counted by a human observer using tanaMove.

### Immunohistochemistry

Two hours after the first 10 trials of the rotarod task, mice were deeply anesthetized with sodium pentobarbital (Somnopentyl, Kyoritsu Seiyaku Co., Tokyo, Japan) and perfused intracardially with 0.9% saline followed by Zamboni solution (4% paraformaldehyde mixed with 0.2% picric acid in phosphate-buffered saline (PBS)) prior to dissecting the whole brains. After postfixation in Zamboni solution overnight at 4°C, the brains were placed into 30% sucrose in PBS, followed by a series of treatments for making embedded frozen brains. The brains were cryosectioned at 60 μm. Adjacent sections containing primary and secondary motor cortex (+1.70 mm to +0.98 mm from the bregma), cingulate cortex (+0.98 mm to −0.22 mm from the bregma), striatum (+0.98 mm to −0.22 mm from the bregma), and cerebellum (−5.68 mm to −6.36 mm from the bregma) were processed for free-floating immunostaining. Free-floating sections were treated with 0.3% H_2_O_2_ in PBS-T (0.1% Triton X-100 in PBS), rinsed three times in PBS-T, and then incubated in PBS-T containing 1.5% normal goat serum and 10% bovine serum albumin for 1 h at room temperature. Sections were incubated with rabbit anti-Fos antibody (1:1,000; sc-52, Santa Cruz Biotechnology, Santa Cruz, CA) or rat anti-Caspr3 (1:2,000; clone 3A2; [12]) in PBS-T with 0.2% bovine serum albumin overnight at 4°C, followed by three rinses in PBS-T. Sections were incubated with biotinylated anti-rabbit IgG (1:250; PK6101, Vector Laboratories, Burlingame, CA) or biotinylated anti-rat IgG (1:250; BA-4001, Vector Laboratories) in PBS-T with 1.5% normal goat serum for 1 h at room temperature and then rinsed several times in PBS-T. The sections were incubated with ABC reagent from the Vectastain Elite ABC kit (Vector Laboratories) for 30 min at room temperature and rinsed with 0.1 M phosphate buffer, pH 7.2. The sections were then incubated in 0.05% 3,3-diaminobenzidine in the presence of 0.01% H_2_O_2_ for 10 min. The sections were finally coverslipped with PermaFlour (Thermo Fisher Scientific Inc., Waltham, MA). Fluorescence images were acquired on an inverted microscope (Biozero BZ-8100; Keyence, Osaka, Japan).

The quantification of c-Fos-positive cells was performed under a 2× objective, which yielded a field of view of 440 × 331 μm. A total of five alternating sections were used for quantifying c-Fos positive cells in each brain region.

### Statistical analysis

Statistical analyses were performed in Statview 5.0J software (SAS Institute, Cary, NC). Before the statistical analysis, we examined the normality and equal variances in our data by using EZR (Saitama, Medical Centre, Jichi Medical University; http://www.jichi.ac.jp/saitama-sct/SaitamaHP.files/statmed.html; [67]). To test the normality of our data, we conducted the Kolmogorov-Smirnov test under the null hypothesis that the data distribution followed a Gaussian distribution. An F-test was then conducted to examine whether the variances of the WT and KO groups were equal. For c-Fos experiments, we used the Bartlett test to examine equal variance among the four groups. When behavioral data followed these assumptions, *t*-tests were used to detect significant differences between Caspr3 WT and KO mice. Otherwise, Mann-Whitney tests, with Bonferroni correction if it is necessary were applied (Table 1, S2 and S8 Tables). Data from the accelerating rotarod test and wheel running test and the quantification of c-Fos-positive cells were analyzed using two-way analysis of variance (ANOVA), and *t*-tests with Bonferroni correction were conducted as post-hoc analyses (Figs 1–3; S4, S6 and S8 Tables). In cases where the data did not follow the normal distribution and show homoscedasticity, the Mann-Whitney test with Bonferroni correction was applied (Figs 1–3; S4, S6 and S8 Tables). p < 0.05 was considered statistically significant. All values are reported as the mean ± SEM.

## Supporting Information

S1 Table Data from a series of behavioral tests. Data obtained from home cage test, open field test, elevated plus maze test, Y-maze test, resident-intruder test, fear conditioning test, startle response test, PPI test, hot plate test, and tail flick test are presented.
(XLSX)

S2 Table Statistics of behavioral data. (XLSX)

S3 Table Data from accelerated rotarod test. (XLSX)

S4 Table Statistics of accelerated rotarod test. (XLSX)

S5 Table Data from wheel running test. (XLSX)

S6 Table Statistics of wheel running test. (XLSX)

S7 Table Data from quantification of c-Fos expressing cells. (XLSX)

S8 Table Statistics of c-Fos expressing cells. (XLSX)
